# A Case of Acute Neck Pain: The Crowned Dens Syndrome

**DOI:** 10.7759/cureus.9555

**Published:** 2020-08-04

**Authors:** Pius E Ojemolon, Ehizogie Edigin, Narender Annapureddy, Augustine Manadan

**Affiliations:** 1 Anatomical Sciences, St. George's University, St. George's, GRD; 2 Internal Medicine, John H. Stroger, Jr. Hospital of Cook County, Chicago, USA; 3 Rheumatology, Vanderbilt University, Nashville, USA; 4 Rheumatology, John H. Stroger, Jr. Hospital of Cook County, Chicago, USA

**Keywords:** pseudogout, arthritis, calcinosis, polyarthralgia, calcium pyrophosphate

## Abstract

Crowned dens syndrome (CDS) is a relatively uncommon presentation of calcium pyrophosphate dihydrate (CPPD) deposition disease that manifests as acute attacks of neck pain with fever, neck rigidity and elevated inflammatory markers related to radiodense deposits of CPPD in ligaments around the odontoid process. We present a case of CDS.

## Introduction

Crowned dens syndrome (CDS) is a relatively uncommon presentation of calcium pyrophosphate dihydrate (CPPD) deposition disease that manifests as fever, neck pain/stiffness and features of systemic inflammation [1-3]. The prevalence of the syndrome is unclear, but it is known to be more common in elderly patients, and the characteristic crown-shaped calcification of the ligaments around the odontoid process (hence the name “crowned dens syndrome”) has been identified in up to 5% of adults over the age of 70 years who present to hospitals with neck pain as the primary complaint. The estimated frequency of periodontoid calcinosis in persons with CPPD deposition disease ranges between 40% and 60% [1,4,5].

CDS has a good prognosis, and symptoms usually subside within a few days to weeks if treatment is started early. Non-steroidal anti-inflammatory drugs (NSAIDs) are the mainstay of therapy. However, CDS is notoriously prone to being misdiagnosed. More common differentials for neck pain usually being considered lead to excessive and often invasive investigations, indiscreet antibacterial or antiviral treatment, prolonged hospital stay and increased health care costs [6-8].

## Case presentation

A 63-year-old Hispanic male presented with polyarthralgia over a one-year period, which caused him to become progressively debilitated and eventually bedridden requiring assistance with basic self-care. He acutely presented to the emergency department with one week of severe neck pain and neck rigidity. Several days after developing neck pain, he developed left shoulder pain and left wrist swelling. Physical examination revealed severely painful and restricted neck range of motion. He also had warm, painful left wrist effusion and painful left shoulder range of motion.

**Figure 1:**
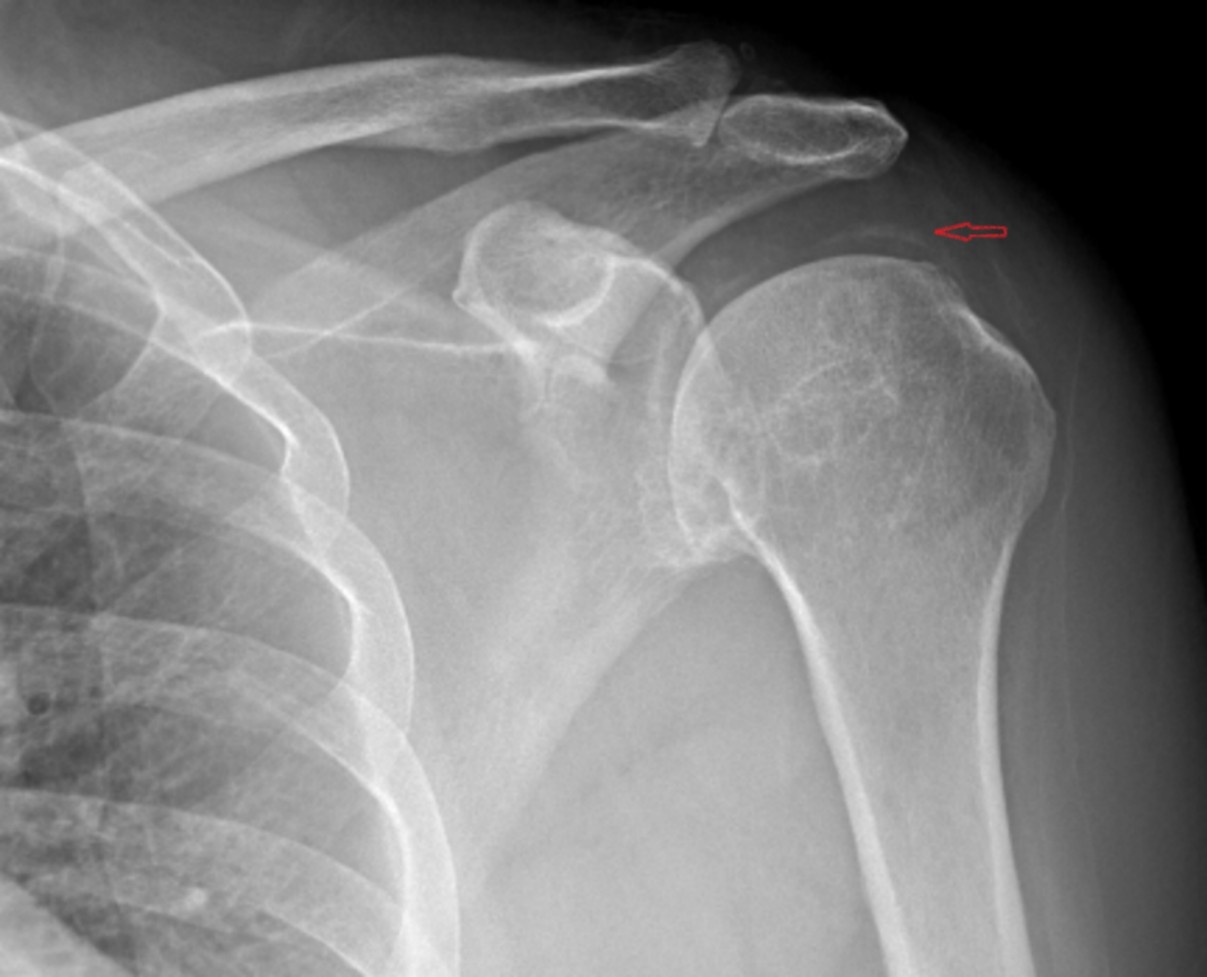
Radiograph showing chondrocalcinosis of the glenohumeral joint space The arrow identifies area of chondrocalcinosis of the glenohumeral joint space.

**Figure 2:**
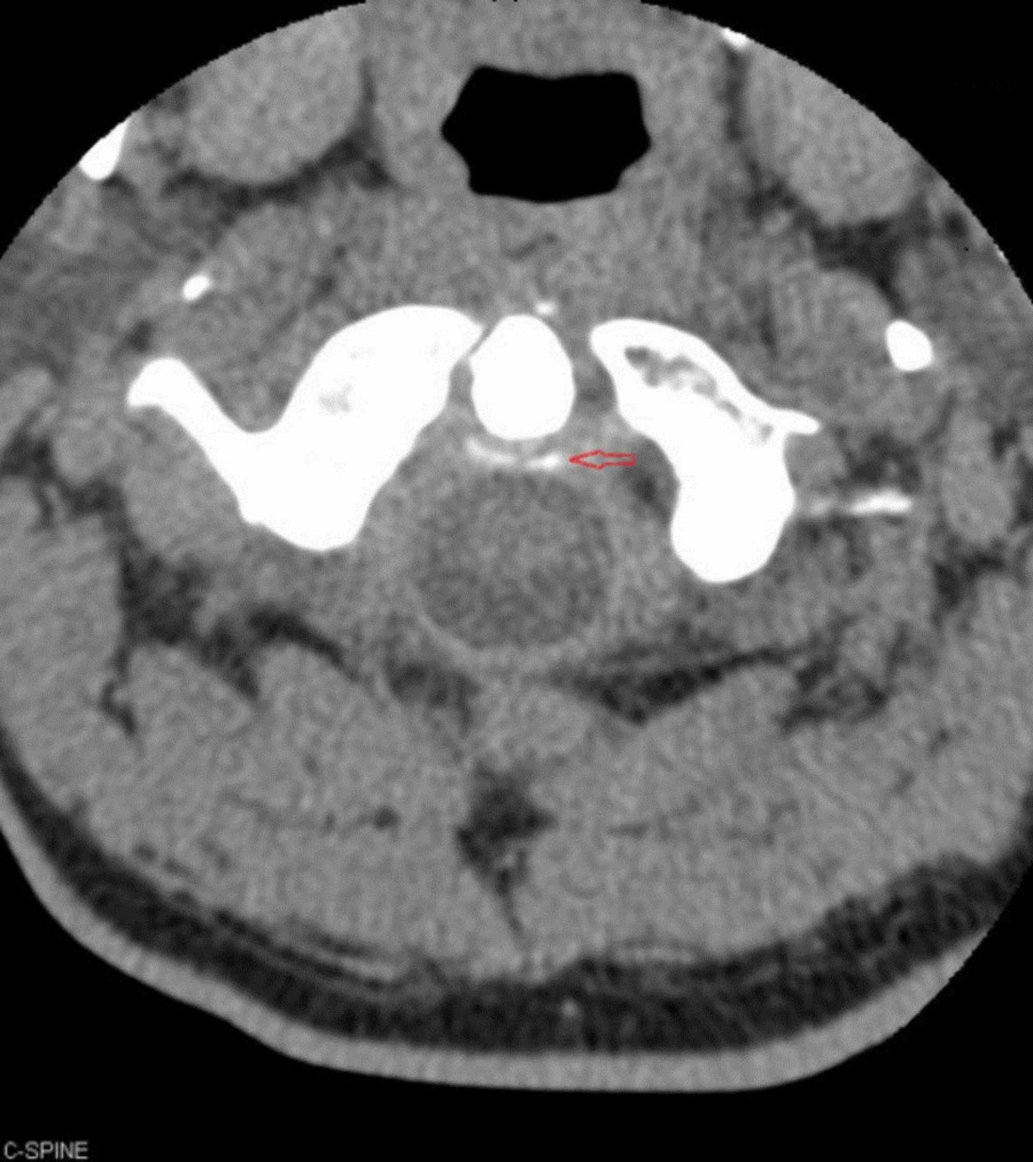
Axial section CT of the neck showing chondrocalcinosis of the transverse ligament of the atlas The arrow highlights area of calcinosis of the transverse ligament posterior to the dens.

**Figure 3:**
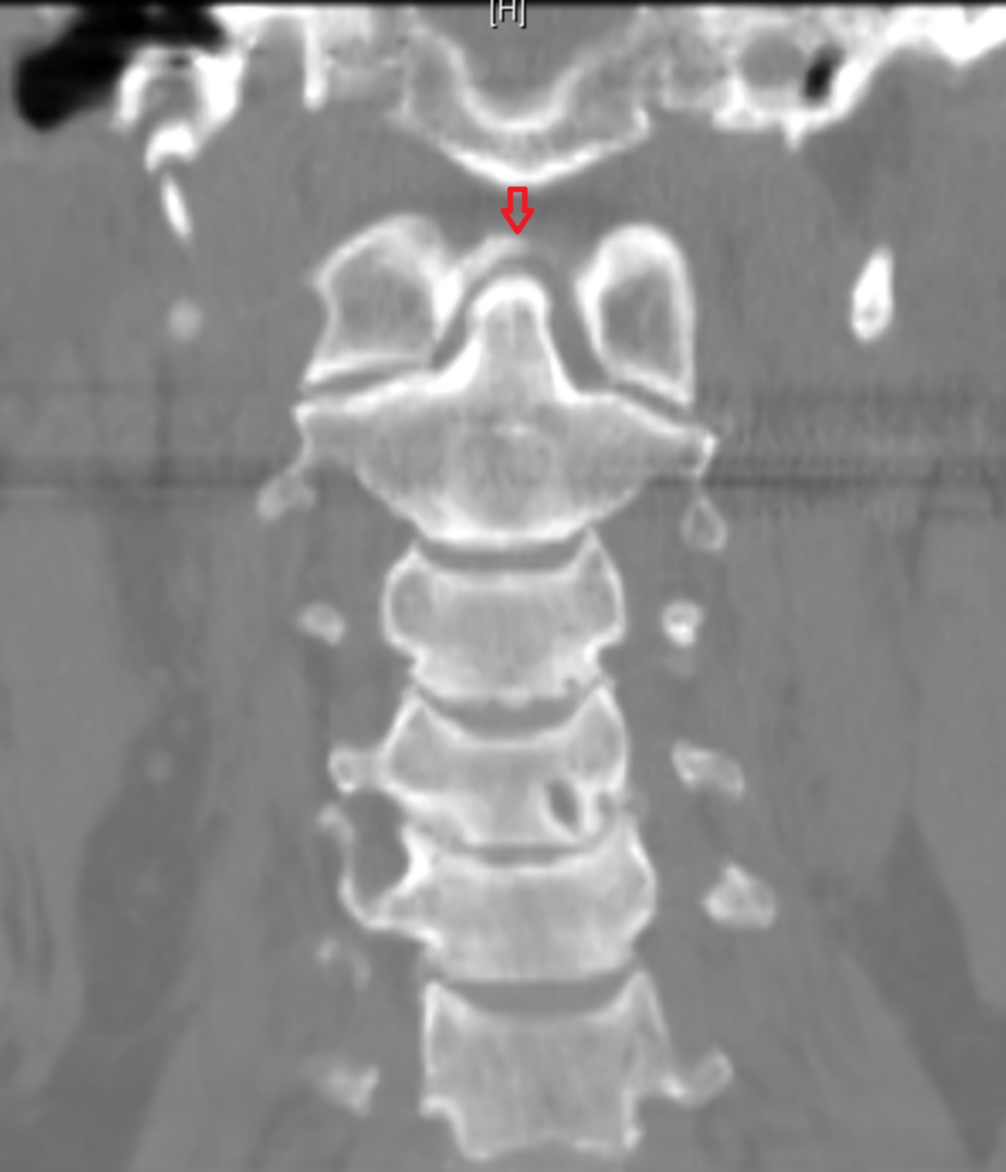
Coronal CT section of the neck showing chondrocalcinosis of the transverse ligament of the atlas The arrow highlights area of calcinosis shaped like a crown or halo around the dens.

Labs revealed an erythrocyte sedimentation rate (ESR) of 52 mm/hr. Aspiration of left wrist yielded one drop of synovial fluid with 2,200 white blood cells/µl but crystal analysis was not performed. Radiographs were obtained that revealed chondrocalcinosis of the glenohumeral joint space (Figure 1). CT of the neck revealed chondrocalcinosis of the transverse ligament of the atlas (Figures 2, 3).

He was given a diagnosis of CDS. In subsequent months, he developed additional attacks of left wrist swelling. Colchicine therapy was started and on follow-up visits, he reported resolution of polyarthralgia and return to normal activity.

## Discussion

CPPD deposition disease, also known as pseudogout, is known to have a variety of clinical presentations depending on the area of calcium crystal deposition [9,10]. The first piece of literature highlighting cervical chondrocalcinosis was published in 1980, and subsequently, Bouvet et al christened it “crowned dens syndrome” in 1985 [11,12]. CDS, a clinical-radiological entity, is fundamentally an attack of pseudogout at the atlantoaxial joint, characterized chiefly by calcification of the cruciform ligament around the odontoid process of the axis vertebra. This causes acute moderate to severe neck pain/stiffness worst at the base of the occiput and fever with raised inflammatory markers (ESR and C-reactive protein) [9-12]. It most commonly occurs in patients aged 60 years and above who may have other manifestations of CPPD deposition disease, including polyarticular arthritis with or without acute attacks of pseudogout affecting the knee, shoulder or wrist joints [13-15].

The exact pathogenesis of CDS is not fully understood, but it is thought to involve chondrocytic transformation of fibroblasts in the ligaments at the atlantoaxial joint with eventual calcium pyrophosphate or hydroxyapatite crystal deposition leading to inflammation of the periodontoid ligaments and surrounding tissues [3,6,13]. Acute or subacute presentations can be misdiagnosed as meningitis, polymyalgia rheumatica, cervical discitis, giant cell arteritis, epidural abscess, rheumatoid arthritis, osteomyelitis, retropharyngeal abscess or a metastatic tumor, and chronic relapsing presentations may be misdiagnosed as cervicogenic neck pain or occipital neuralgia. Rarely, the inflammatory process in cases of chronic CDS can progressively erode the atlantoaxial ligaments and the dens leading to atlantoaxial instability, cervical spinal cord compression and progressive quadriparesis. Misdiagnosis of CDS may result in inappropriate invasive investigations such as lumbar puncture and subsequent treatment with parenteral broad-spectrum antimicrobial therapy or prolonged external neck immobilization [1,5,8-10].

While the diagnosis should be suspected in elderly patients with the typical clinical picture, unenhanced CT imaging of the atlantoaxial joint is the gold standard for diagnosing CDS, with tomograms (particularly coronal sections) showing calcifications in the periodontoid ligaments between the dens and the atlas. Neck radiographs do not delineate these calcifications clearly. CT may also show soft tissue thickening and subchondral bone cyst formation and help rule out alternative causes of neck pain. MRI and nuclear medicine techniques, such as integrated single-photon emission computed tomography (SPECT) imaging, may be useful in ruling out differential diagnoses but they do not highlight calcifications as well as CT imaging [1,7,9].

CDS is fairly amenable to treatment once correctly diagnosed, and there is often a marked improvement in symptoms within days to weeks of commencement of therapy. First-line therapy for CDS parallels that for pseudogout and typically involves use of NSAIDs. Colchicine is also gaining popularity as a therapeutic choice. Patients who cannot tolerate NSAIDs or colchicine are usually treated with low-dose corticosteroids [6,10]. Other risk factors for CPPD disease, such as hypomagnesemia, hyperparathyroidism, hypophosphatemia and hemochromatosis, should also be addressed if present [9,16].

## Conclusions

CDS is an uncommon condition that is fairly amenable to treatment but is often mistaken for more common causes of fever and neck pain. Hence, in elderly patients with sudden onset neck pain/stiffness, fever and features of systemic inflammation, CDS should be considered in the differential diagnoses.
